# Supplementary material for: Cluster Analysis Identifies Distinct Patterns of T-Cell and Humoral Immune Responses Evolution Following a Third Dose of SARS-CoV-2 Vaccine in People Living with HIV
Source: Viruses. 2023 Jun 26;15(7):1435. doi: 10.3390/v15071435 (PMC10383048; doi:10.3390/v15071435)
Supplement: Supplementary file 1 [file viruses-15-01435-s001.zip › viruses-2337606-supplementary.pdf]

Supplementary files

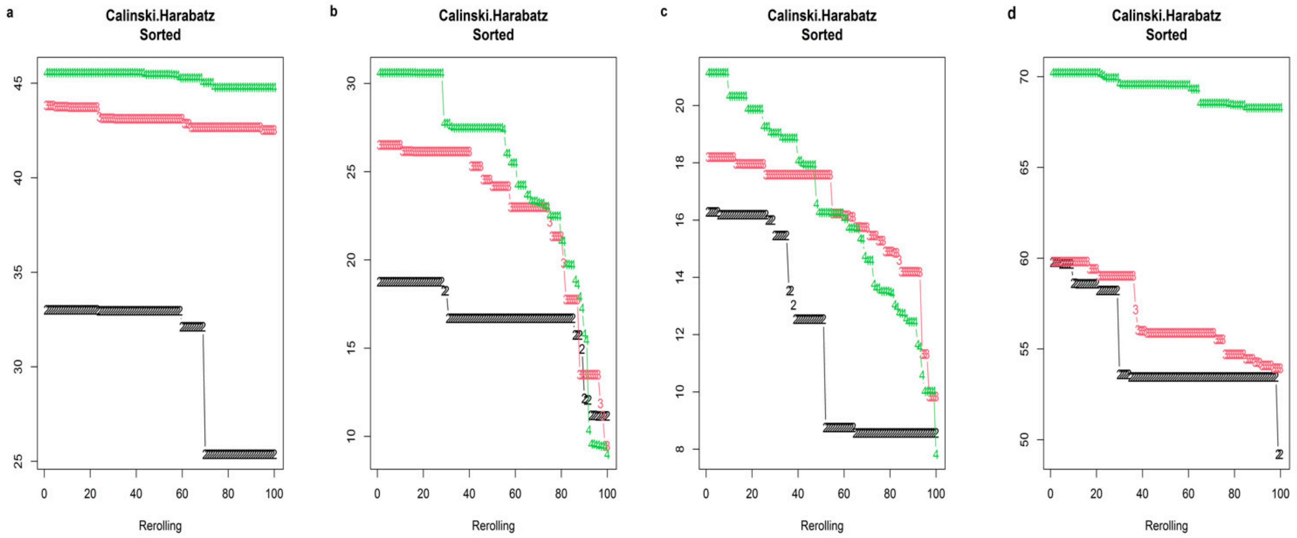

Figure S1. Clustering of T-cell and humoral immune responses evolution: Calinski and Harabatz criterion maximization. Clustering with 100 runs for each cluster; 2 (in black), 3 (in red), and 4 (in green) clusters. **a.** Clustering among SARS-CoV-2 naïve individuals (n = 68), **b.** clustering among SARS-CoV-2 naïve PLWH (n = 39), **c.** clustering among SARS-CoV-2 naïve HCWs (n = 29). **d.** clustering among all individuals either SARS-CoV-2 experienced and naïve individuals (n=131).

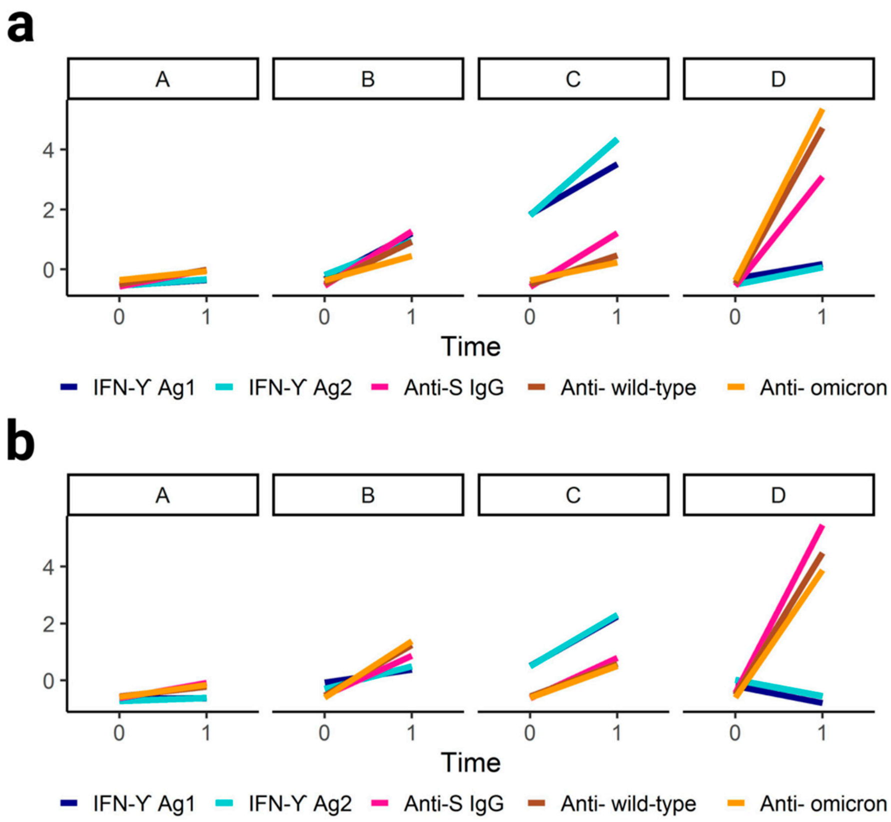

Figure S2. Clustering of T-cell and humoral immune responses evolution. IFN-Ɣ Ag1, IFN-Ɣ Ag2, Anti-S IgG, and neutralising antibody titers against Wild type and Omicron variants patterns of evolution in each of the four clusters (a) among SARS-CoV-2 naïve PLWH (cluster A: n = 21, cluster B: n = 10, cluster C: n = 2, cluster D: n = 2), (b) among SARS-CoV-2 naïve HCWs (cluster A: n = 13, cluster B: n = 9, cluster C: n = 5, cluster D: n = 1).

Table S1. IFN-ɣ Ag1 and Ag2, Anti-S IgG, and neutralizing antibody titers against Wild-type and Omicron variants in each cluster.

Variable	Cluster A n = 86	Cluster B n = 21	Cluster C n = 10	Cluster D n = 3
**IFN-ɣ Ag1**				
T0	0.35 (0.11-0.72)	1.6 (1.1-3.9)	0.32 (0.14-0.92)	9.9 (9.6-10.0)
T1	0.69 (0.31-1.4)	4.4 (3.3-5.5)	1.1 (0.50-1.4)	10.0 (9.9-10.0)
**IFN-ɣ Ag2**				
T0	0.42 (0.15-1.0)	2.2 (1.9-4.8)	0.24 (0.17-0.90)	9.8 (9.7-9.9)
T1	0.91 (0.44-1.9)	6.1 (5.2-6.9)	1.7 (0.65-2.3)	10.0 (9.9-10.0)
**Anti-S IgG**				
T0	543 (304-1325)	1350 (315-2040)	927 (471-1705)	17500 (8963-24300)
T1	5270 (2882-9958)	11100 (3270-14900)	30900 (18300-38525)	46400 (31850-50750)
**Neutralizing antibody titers against wild type**				
T0	80 (20-160)	160 (40-640)	80 (80-160)	5120 (2580-5120)
T1	1280 (640-2560)	2560 (640-5120)	10240 (10240-10240)	10240 (10240-10240)
**Neutralizing antibody titers against omicron**				
T0	<20 (<20-<20)	<20 (<20-40.0)	<20 (<20-15)	320 (160-800)
T1	160 (80-320)	160 (80-320)	1280 (800-2240)	2560 (1600-3840)

Results are expressed as Median (Q1-Q3).

Table S2. Background characteristics of individuals in each cluster.

Variable	Cluster A n = 86	Cluster B n = 21	Cluster C n = 10	Cluster D n = 3	Comparison p-value
Male sex	41 (47.7)	8 (38.1)	1 (10.0)	2 (66.7)	0.083
Age (Years)	43.7 ± 10.3	42.1 ± 10.1	51.0 ± 11.0	52.3 ± 16.0	0.078
18-29	5 (5.8)	0 (0.0)	1 (10.0)	0 (0.0)	
30-39	31 (36.1)	12 (57.1)	0 (0.0)	1 (33.3)	
40-49	23 (26.8)	3 (14.3)	2 (20.0)	0 (0.0)	
50-59	21 (24.4)	4 (19.1)	6 (60.0)	0 (0.0)	
≥60	6 (6.9)	2 (9.5)	1 (10.0)	2 (66.7)	
BMI (kg/m^2)	25.9 ± 5.1, n = 85	26.6 ± 7.4	31.3 ± 9.3	29.0 ± 3.8	0.052
< (<18.5)	2 (2.3)	0 (0.0)	0 (0.0)	0 (0.0)	
Normal range (18.5-24.9)	37 (43.5)	10 (47.6)	1 (10.0)	0 (0.0)	
Overweight (25-29.9)	32 (37.7)	8 (38.1)	5 (50.0)	2 (66.7)	
Obese (≥30)	14 (16.5)	3 (14.3)	4 (40.0)	1 (33.3)	
Ethicity, n = 71					0.0001
Caucasian	32 (59.3)	0 (0.0)	0 (0.0)	0 (0.0)	
African	19 (35.2)	6 (75.0)	6 (100.0)	3 (100.0)	
Other	3 (5.5)	2 (25.0)	0 (0.0)	0 (100.0)	
Medical history					
Diabetes mellitus	2 (2.3)	0 (0.0)	1 (10.0)	1 (33.3)	-
Hypertension	13 (15.1)	3 (14.3)	5 (50.0)	0 (0.0)	0.066
Heart failure coronary artery disease	2 (2.3)	0 (0.0)	0 (0.0)	0 (0.0)	-
Stroke	0 (0.0)	1 (4.8)	0 (0.0)	0 (0.0)	-
Liver disease	1 (1.2)	0 (0.0)	0 (0.0)	0 (0.0)	-
Kidney disease	0 (0.0)	0 (0.0)	0 (0.0)	0 (0.0)	-
Chronic lung disease	1 (1.2)	0 (0.0)	0 (0.0)	0 (0.0)	-
Asthma	0 (0.0)	0 (0.0)	3 (30.0)	0 (0.0)	-
Autoimmune disease	2 (2.3)	0 (0.0)	0 (0.0)	0 (0.0)	-
Hematological cancer	1 (1.2)	0 (0.0)	0 (0.0)	0 (0.0)	-
Non hematological cancer	8 (9.3)	0 (0.0)	1 (10.0)	1 (33.3)	-
Solid-organ/cell transplantation	0 (0.0)	0 (0.0)	0 (0.0)	0 (0.0)	-
HCWs	32 (37.2)	13 (61.9)	4 (40.0)	0 (0.0)	0.10
PLWH	54 (62.8)	8 (38.1)	6 (60.0)	3 (100.0)	
Prior AIDS diagnosis	21 (24.4), n = 54	3 (14.3), n = 8	1 (10.0), n = 6	0 (0.0)	0.52
Time at T0 since HIV diagnosis (years)	11 (5-18)	11 (6-14)	13 (8-20)	14 (9-16)	0.90
Time on ART (years),	10.4 ± 7.4	8.3 ± 4.7	13.4 ± 7.3	10.3 ± 6.5	0.61
Nadir CD4+T cell count per µL	282 (143-524)	250 (93-387)	344 (267-408)	335(274-379)	0.86
Last CD4+T cell count per µL (2021 or 2022)	734(601-932)	603 (401-923)	761 (647-781)	807 (690-1058)	0.75
CD4/CD8 ratio	0.93 (0.63-1.4)	0.96 (0.49-1.1)	1.0 (0.83-1.3)	0.96 (0.75-1.1)	0.74
Last plasma viral load copies/mL	<20 (<20-<20)	<20 (<20-<20)	<20 (<20-<20)	<20 (<20-<20)	0.88
SARS-COV-2 infection status					0.011
Naïve	51 (59.3)	9 (42.9)	3 (30.0)	0 (0.0)	
Experienced before T0	28 (32.6)	11 (52.4)	3 (30.0)	2 (66.7)	
Experienced between T0 and T1	7 (8.1)	1 (4.8)	4 (40.0)	1 (33.3)	
First vaccine dose					0.46
BNT162b2 mRNA (Pfizer)	79 (91.9)	19 (90.4)	9 (90.0)	3 (100.0)	
mRNA-1273 (Moderna)	1 (1.2)	1 (4.8)	1 (10.0)	0 (0.0)	
ChAdOx1-S (Astra Zeneca)	6 (6.9)	1 (4.8)	0 (0.0)	0 (0.0)	
Second vaccine dose					0.46
BNT162b2 mRNA (Pfizer)	79 (91.9)	19 (90.4)	9 (90.0)	3 (100.0)	
mRNA-1273 (Moderna)	1 (1.2)	1 (4.8)	1 (10.0)	0 (0.0)	
ChAdOx1-S (Astra Zeneca)	6 (6.9)	1 (4.8)	0 (0.0)	0 (0.0)	
Third vaccine dose					0.48
BNT162b2 mRNA (Pfizer)	60 (71.4)	17 (81.0)	6 (60.0)	3 (100.0)	
mRNA-1273 (Moderna)	26 (28.6)	4 (19.0)	4 (40.0)	0 (0.0)	
Time between first and second vaccine dose (weeks)	4.4 (3.0-5.0)	3.1 (3.0-4.0)	3·9 (3·0-5.0)	3.1 (3.1-4.1)	0.35
Time between second vaccine dose and sample at T0 (weeks)	24 (24-26)	24 (24-25)	24 (23-27)	26 (22-32)	0.93
Time between second and third vaccine dose (weeks)	30 (25-38)	35 (30-39)	32 (27-38)	30 (26-34)	0.20
Time between third vaccine dose and sample at T1 (weeks)	3.9 (2.9-4.8)	4.1 (2.9-4.7)	3.4 (2.8-4.9)	3.3 (3.1-4.0)	0.92

Time between T0 and T1 (weeks)	6 (4-19)	18 (8-19)	7 (7-18)	7 (6-7)	0.18

Results are expressed as N (%), mean ± SD, or Median (Q1-Q3) as appropriate, and p-value of Fisher exact test, ANOVA or Kruskal-Wallis test respectively.

Table S3. IFN-ɣ Ag1 and Ag2, Anti-S IgG, and neutralizing antibody titers against Wild-type and Omicron variants in each cluster of SARS-CoV-2 naïve population.

**Variable**	**Cluster A**<br>**n = 35**	**Cluster B**<br>**n = 18**	**Cluster C**<br>**n = 7**	**Cluster D**<br>**n = 3**
**IFN-ɣ Ag1**				
T0	0.12 (0.050-0.39)	0.069 (0.21-1.3)	1.6 (1.4-2.8)	0.48 (0.39-0.78)
T1	0.35 (0.16-0.55)	1.8 (1.4-2.2)	3.9 (3.2-5.0)	0.49 (0.42-0.93)
**IFN-ɣ Ag2**				
T0	0.16 (0.069-0.47)	1.2 (0.39-1.7)	2.2 (2.0-4.6)	0.25 (0.24-1.2)
T1	0.48 (0.23-0.88)	2.8 (1.9-3.3)	6.1 (5.8-7.2)	0.97 (0.76-1.2)
**Anti-S IgG**				
T0	308 (146-515)	665 (419-1157)	313 (259-562)	460 (453-1220)
T1	4100 (2190-5860)	11600 (7655-14975)	12700 (8930-15450)	37500 (28050-42150)
**Neutralizing antibody titers against wild type**				
T0	20 (<20-40)	80 (40-140)	40 (<20-60)	80 (80-120)
T1	640 (320-1280)	2560 (2560-5120)	1280 (640-3840)	10240 (10240-10240)
**Neutralizing antibody titers against omicron**				
T0	<20 (<20-<20)	<20 (<20-<20)	<20 (<20-<20)	<20 (<20-<20)
T1	80 (40-120)	320 (160-320)	160 (80-240)	1280 (960-1920)

Results are expressed as Median (Q1-Q3).

Table S4. Background characteristics of SARS-CoV-2 naïve individuals in each cluster.

Variable	Cluster A<br>n = 35	Cluster B<br>n = 18	Cluster C<br>n = 7	Cluster D<br>n = 3	Comparison<br>p-value
Male sex	16 (45.7)	13 (72.2)	4 (57.1)	2 (66.7)	0.30
Age (Years)	43.5 ± 10.9	41.8 ± 8.7	42.4 ± 11.8	41.0 ± 16.1	0.94
18-29	3 (8.6)	0 (0.0)	0 (0.0)	1 (33.3)	
30-39	13 (37.1)	8 (44.4)	4 (57.1)	0 (0.0)	
40-49	7 (20.0)	6 (33.3)	0 (0.0)	1 (33.3)	
50-59	10 (28.6)	3 (16.7)	2 (28.6)	1 (33.3)	
≥60	2 (5.7)	1 (5.6)	1 (14.3)	0 (0.0)	
BMI (kg/m^2)	25.3 ± 4.0, N = 34	25.2 ± 3.6	25.0 ± 2.8	37.5 ± 17.3	0.73
< (<18.5)	1 (2.9)	0 (0.0)	0 (0.0)	0 (0.0)	
Normal range (18.5-24.9)	14 (41.2)	8 (44.4)	2 (28.6)	1 (33.3)	
Overweight (25-29.9)	17 (50.0)	8 (44.4)	5 (71.4)	0 (0.0)	
Obese (≥30)	2 (5.9)	2 (11.1)	0 (0.0)	2 (66.7)	
Ethicity	N = 22	N = 9	N = 2	N = 2	-
Caucasian	16 (72.7)	3 (33.3)	0 (0.0)	0 (0.0)	
African	3 (13.6)	6 (66.7)	2 (100.0)	2 (100.0)	
Other or unknown	3 (13.6)	0 (0.0)	0 (0.0)	0 (0.0)	
Medical history					
Diabetes mellitus	0 (00)	1 (5.6)	0 (00)	0 (00)	-
Hypertension	3 (8.6)	2 (11.1)	1 (14.3)	2 (66.7)	0.078
Heart failure coronary artery disease	1 (2.9)	0 (0.0)	0 (0.0)	0 (0.0)	-
Stroke	0 (0.0)	0 (0.0)	0 (0.0)	0 (0.0)	-
Liver disease	0 (0.0)	0 (0.0)	0 (0.0)	0 (0.0)	-
Kidney disease	0 (0.0)	0 (0.0)	0 (0.0)	0 (0.0)	-
Chronic lung disease	0 (0.0)	0 (0.0)	0 (0.0)	0 (0.0)	-
Asthma	0 (0.0)	0 (0.0)	0 (0.0)	1 (33.3)	-
Autoimmune disease	2 (5.7)	0 (0.0)	0 (0.0)	0 (0.0)	-
Hematological cancer	1 (2.9)	0 (0.0)	0 (0.0)	0 (0.0)	-
Non hematological cancer	3 (8.6)	2 (11.1)	0 (0.0)	1 (33.3)	-
Solid-organ/cell transplantation	0 (0.0)	0 (0.0)	0 (0.0)	0 (0.0)	-
HCW	13 (37.1)	9 (50.0)	5 (71.4)	1 (33.3)	0.38
PLWH	22 (62.9)	9 (50.0)	2 (28.6)	2 (66.7)	
Prior AIDS diagnosis	9 (40.9), n = 22	3 (33.3), n = 9	1 (50.0), n = 2	1 (50.0), n = 2	1.0
Time at T0 since HIV diagnosis (years)	11 (5-14)	15 (7-18)	13 (10-16)	19 (13-25)	0.88
Time on ART (years),	9.9 ± 7.3	11.3 ± 7.2	7.7 ± 3.1	15.3 ± 11.4	0.71
Nadir CD4+T cell count per µL	284 (136-480)	208 (199-416)	182 (146-218)	315(263-367)	0.88
Last CD4+T cell count per µL (2021 or 2022)	707 (552-842)	842 (600-1159)	513 (432-595)	780 (778-781)	0.46
CD4/CD8 ratio	0.89 (0.65-1.3)	1.3 (0.94-2.0)	0.86 (0.70-1.0)	1.2 (1.2-1.3)	0.40
Last plasma viral load copies/mL	<20 (<20-<20)	<20 (<20-<20)	33 (21--44)	<20 (<20-<20)	0.43
SARS-COV-2 infection status					-
Naïve	35 (100.0)	18 (100.0)	7 (100.0)	3 (100.0)	
Experienced before T0	0 (0.0)	0 (0.0)	0 (0.0)	0 (0.0)	
Experienced between T0 and T1	0 (0.0)	0 (0.0)	0 (0.0)	0 (0.0)	
First vaccine dose					0.24
BNT162b2 mRNA (Pfizer)	34 (97.1)	15 (83.3)	7 (100.0)	3 (100.0)	
mRNA-1273 (Moderna)	0 (0.0)	1 (5.6)	0 (0.0)	0 (0.0)	
ChAdOx1-S (Astra Zeneca)	1 (2.9)	2 (11.1)	0 (0.0)	0 (0.0)	
Second vaccine dose					0.24
BNT162b2 mRNA (Pfizer)	34 (97.1)	15 (83.3)	7 (100.0)	3 (100.0)	
mRNA-1273 (Moderna)	0 (0.0)	1 (5.6)	0 (0.0)	0 (0.0)	
ChAdOx1-S (Astra Zeneca)	1 (2.9)	2 (11.1)	0 (0.0)	0 (0.0)	
Third vaccine dose					0.41
BNT162b2 mRNA (Pfizer)	28 (80.0)	11 (61.1)	6 (85.7)	2 (66.7)	
mRNA-1273 (Moderna)	7 (20.0)	7 (38.9)	1 (14.3)	1 (33.3)	
Time between first and second vaccine dose (weeks)	4.9 (3.0-5.0)	3.1 (3.0-4.8)	3.1 (3.0-3.7)	4.3 (3.9-4.9)	0.23
Time between second vaccine dose and sample at T0 (weeks)	24 (24-25)	24 (24-26)	24 (24-26)	23 (22-25)	0.48
Time between second and third vaccine dose (weeks)	27 (25-37)	34 (30-39)	38 (36-39)	31 (28-32)	0.073

Time between third vaccine dose and sample at T1 (weeks)	4.0 (2.9-4.8)	4.2 (3.1-5.1)	4.1 (3.9-4.7)	3.7 (3.2-5.9)	0.88
Time between T0 and T1 (weeks)	6 (4-19)	11 (4-19)	18 (13-19)	7 (7-13)	0.62

Results are expressed as N (%), mean ± SD, or Median (Q1-Q3) as appropriate, and p-value of Fisher exact test, ANOVA or Kruskal-Wallis test.
